# Supplementary material for: Readability of Commonly Used Quality of Life Outcome Measures for Youth Self-Report
Source: Int J Environ Res Public Health. 2022 Aug 3;19(15):9555. doi: 10.3390/ijerph19159555 (PMC9367855; doi:10.3390/ijerph19159555)
Supplement: Supplementary file 1 [file ijerph-19-09555-s001.zip › Supplement File S1 - Text Characteristics - 2022-06-01.pdf]

Readability of Commonly Used Quality of Life Outcome Measures for Youth Self-Report

Karolin R. Krause , Jenna Jacob *, Peter Szatmari and Daniel Hayes

Supplement File S1:

Text Characteristics of the Included QOL Measures

Table S1. Text Characteristics of the Included QOL Measures

			Instructions						Items					
Measure	Ages	Length	Letters	Words	Sentences	Av. Sentence Length	Av. Word Length	Difficult Words [a]	Letters	Words	Sentences	Av. Sentence Length	Av. Word Length	Difficult Words [a]
AQOL-6D Adolescent Instrument	12-18 years	20 items	363	73	4	18.3	5.0	25%	4430	1000	123	8.1	4.5	18%
CHU-9D	7-17 years	9 items	369	95	11	8.6	3.9	3%	1478	363	53	6.8	4.1	17%
EQ-5D-Y	8-15 years	6 items	293	77	7	11.0	3.9	14%	697	155	22	7.0	4.5	19%
KIDSCREEN – *KIDSCREEN-52*	8-18 years	52 items	447	109	11	9.9	4.1	5%	2203	527	61	8.6	4.2	9%
KIDSCREEN – *KIDSCREEN-27*	8-18 years	27 items	447	109	11	9.9	4.1	5%	1177	287	30	9.6	4.1	10%
KIDSCREEN – *KIDSCREEN-10*	8-18 years	10 items	447	109	11	9.9	4.1	5%	350	89	12	7.4	3.9	4%
KINDL[R] – *Kid-KINDL[R]*	7-13 years	24 items	693	165	18	9.2	4.2	6%	656	173	25	6.9	3.8	12%
KINDL[R] – *Kiddo-KINDL[R]*	14-17 years	24 items	739	176	19	9.3	4.2	6%	633	169	24	7.0	3.7	12%
PedsQL 4.0 *Generic Core Scales – Child*	8-12 years	23 items	320	91	8	11.4	3.6	14%	805	218	29	7.5	3.7	8%
PedsQL 4.0 *SF15 Generic Core Scales – Child*	8-12 years	15 items	320	91	8	11.4	3.6	14%	577	154	21	7.3	3.7	10%
PedsQL 4.0 *Generic Core Scales – Teen*	13-18 years	23 items	320	91	8	11.4	3.6	14%	797	215	29	7.4	3.7	10%
PedsQL 4.0 *SF15 Generic Core Scales – Teen*	13-18 years	15 items	320	91	8	11.4	3.6	14%	580	154	21	7.3	3.8	12%
PQ-LES-Q	6-17 years	15 items	–	–	–	–	–	–	309	65	14	4.6	4.75	9%
PROMIS Global Health 7	8-17 years	7 items	80	17	2	8.5	4.8	41%	344	80	7	11.4	4.3	10%
PROMIS Global Health 7 +2	8-17 years	9 items	80	17	2	8.5	4.8	41%	404	98	9	10.9	4.13	11%
QOLP-AV	14-20 years	72 items	5327	1253	82	15.3	4.3	24%	4319	949	172	5.5	4.6	24%
TACQOL	8-15 years	63 items	846	199	18	11.1	4.3	11%	2288	473	73	6.5	4.8	22%
YQOL – *YQOL-R*	11-18 years	57 items	1270	270	23	11.7	4.7	14%	3777	891	71	12.5	4.3	9%
YQOL – *YQOL-SF*	11-18 years	16 items	1004	216	18	12.0	4.6	14%	860	205	16	12.8	4.2	8%
16D	12-15 years	16 items	192	43	4	10.8	4.5	12%	4399	1024	100	10.2	4.3	26%
17D	8-11 years	17 items	160	35	4	8.8	4.6	9%	3945	970	121	8.0	4.1	12%
Overall mean (SD)			701.9 (1131.5)	166.4 (264.2)	13.9 (17.2)	10.9 (2.4)	4.2 (0.4)	15% (10.8)	1668.0 (1536.2)	393.3 (349.7)	49.2 (45.5)	8.3 (2.2)	4.1 (0.3)	13% (5.8)

Note: AQOL-6D: Assessment of Quality of Life; CHU-9D: Child Health Utility Index 9D; EQ-5D-Y: EuroQol Five Dimensions Health for Youth; KINDL[R]: KINDer Lebensqualitätsfragebogen; PedsQL 4.0: Pediatric Quality of Life Inventory Generic Core Scales; PQ-LES-Q: The Pediatric Quality of Life Enjoyment and Satisfaction Questionnaire; PROMIS: the Patient Reported Outcomes Measurement Information System; QOLP-AV: Quality of Life Profile - Adolescent Version; TACQOL: TNO AZL Children's Quality of Life; YQOL: Youth Quality of Life Instrument; YQOL-R: Youth Quality of Life Instrument – Research Version; YQOL-SF: Youth Quality of Life Instrument – Short Form; SF15: Short Form 15.

[a] Percentage of difficult vocabulary as indicated by the Dale-Chall formula in R KoRpus.
