# Supplementary material for: Readability of Commonly Used Quality of Life Outcome Measures for Youth Self-Report
Source: Int J Environ Res Public Health. 2022 Aug 3;19(15):9555. doi: 10.3390/ijerph19159555 (PMC9367855; doi:10.3390/ijerph19159555)
Supplement: Supplementary file 1 [file ijerph-19-09555-s001.zip › Supplement File S2 - Readability for Demographic Question Sets - 2022-06-01.pdf]

Readability of Commonly Used Quality of Life Outcome Measures for Youth Self-Report

Karolin R. Krause , Jenna Jacob *, Peter Szatmari and Daniel Hayes

Supplement File S2:

Text Characteristics and Readability of Demographic Question Sets

Table S2. Text Characteristics and Readability of Demographic Question Sets

				Text Characteristics						Items					
Measure	Ages	Length	Citations	Letters	Words	Sentences	Av. Sentence Length	Av. Word Length	Difficult Words [a]	CLI	DC	FK	FOG	FCST	Mean
KINDL^{R} – *Kid-KINDL^{R}* [47, 48]	7-13 years	24 items	1218	64	21	4	5.3	3.0	14%	N/A	10.2	4.4	7.1	11.4	8.3
KINDL^{R} – *Kiddo-KINDL^{R}* [47, 48]	14-17 years	24 items	1218	64	21	4	5.3	3.0	14%	N/A	10.2	4.4	7.1	11.4	8.3
YQOL – *YQOL-R* [54]	11-18 years	57 items	282	531	123	22	5.6	4.3	11%	9.3	9.9	10.5	7.6	13.5	10.2
YQOL – *YQOL-SF* [54]	11-18 years	16 items	282	348	80	14	5.7	4.4	10%	9.6	9.8	10.6	8.3	13.8	11.4
Overall mean				251.8	61.3	11	5.5	3.7	12.3	9.5	7.5	6.4	6.8	12.5	9.55

Note: KINDL^{R}: KINDer Lebensqualitätsfragebogen; YQOL: Youth Quality of Life Instrument; YQOL-R: Youth Quality of Life Instrument – Research Version; YQOL-SF: Youth Quality of Life Instrument – Short Form; SF15: Short Form 15.
