# Supplementary material for: Readability of Commonly Used Quality of Life Outcome Measures for Youth Self-Report
Source: Int J Environ Res Public Health. 2022 Aug 3;19(15):9555. doi: 10.3390/ijerph19159555 (PMC9367855; doi:10.3390/ijerph19159555)
Supplement: Supplementary file 1 [file ijerph-19-09555-s001.zip › Supplement File S3 - R code - 2022-06-01.pdf]

Readability of Commonly Used Quality of Life Outcome Measures for Youth Self-Report

Karolin R. Krause , Jenna Jacob *, Peter Szatmari and Daniel Hayes

Supplement File S3:

KoRpus Analysis Code (Example for one measure's instructions)

---

library(koRpus) library(koRpus.lang.en)

#set the KoRpus environment set.kRp.env(lang="en") set.kRp.env(lang="TRUE") set.kRp.env(TT.cmd="C:\TreeTagger\bin\tag-english.bat", lang="en", preset="en", treetagger="manual", format="file", TT.tknz=TRUE, encoding="UTF-8")

#PedsQL Instruction pedsql_instructions.text <- treetag("Text_PedsQL_Instruction_13-18.txt", treetagger="manual", lang="en", sentc.end = c(".", "!", "?"), TT.options=list(path="c:/Treetagger", preset="en"), doc_id="sample")

describe(pedsql_instructions.text)

(hyph.txt.en <- hyphen(pedsql_instructions.text)) hyph.txt.en head(hyphenText(hyph.txt.en)) hyph.txt.en <- correct.hyph(hyph.txt.en, word="Over", hyphen="O-ver") hyph.txt.en <- correct.hyph(hyph.txt.en, word="along", hyphen="a-long") hyph.txt.en <- correct.hyph(hyph.txt.en, word="asleep", hyphen="a-sleep") hyph.txt.en <- correct.hyph(hyph.txt.en, word="tired", hyphen="ti-red") hyph.txt.en <- correct.hyph(hyph.txt.en, word="over-eat-ing", hyphen="ov-er-eat-ing") hyph.txt.en <- correct.hyph(hyph.txt.en, word="About", hyphen="A-bout") hyph.txt.en <- correct.hyph(hyph.txt.en, word="about", hyphen="a-bout") hyph.txt.en <- correct.hyph(hyph.txt.en, word="ac-ti-v-i-ties", hyphen="ac-ti-vi-ties") hyph.txt.en <- correct.hyph(hyph.txt.en, word="ac-tiv-i-ty", hyphen="ac-ti-vi-ty") hyph.txt.en <- correct.hyph(hyph.txt.en, word="heavy", hyphen="hea-vy") hyph.txt.en <- correct.hyph(hyph.txt.en, word="afraid", hyphen="af-raid") hyph.txt.en <- correct.hyph(hyph.txt.en, word="cho-res", hyphen="chores") hyph.txt.en <- correct.hyph(hyph.txt.en, word="over", hyphen="o-ver") hyph.txt.en <- correct.hyph(hyph.txt.en, word="tummyache", hyphen="tum-my-ache") hyph.txt.en <- correct.hyph(hyph.txt.en, word="alone", hyphen="a-lone") hyph.txt.en <- correct.hyph(hyph.txt.en, word="id-eas", hyphen="i-de-as") hyph.txt.en <- correct.hyph(hyph.txt.en, word="gra-des", hyphen="grades") hyph.txt.en <- correct.hyph(hyph.txt.en, word="many", hyphen="ma-ny") hyph.txt.en <- correct.hyph(hyph.txt.en, word="co-mes", hyphen="comes") hyph.txt.en <- correct.hyph(hyph.txt.en, word="an-y-body", hyphen="an-y-bod-y") hyph.txt.en <- correct.hyph(hyph.txt.en, word="Ac-ti-v-i-ties", hyphen="Ac-ti-vi-ties") hyph.txt.en <- correct.hyph(hyph.txt.en, word="alive", hyphen="a-live") hyph.txt.en <- correct.hyph(hyph.txt.en, word="an-y-th-ing", hyphen="an-y-thing") hyph.txt.en <- correct.hyph(hyph.txt.en, word="body", hyphen="bo-dy") hyph.txt.en <-

correct.hyph(hyph.txt.en, word=“enough”, hyphen=“e-nough”) hyph.txt.en <-
correct.hyph(hyph.txt.en, word=“able”, hyphen=“a-ble”) hyph.txt.en <-
correct.hyph(hyph.txt.en, word=“imag-ine”, hyphen=“i-mag-ine”) hyph.txt.en <-
correct.hyph(hyph.txt.en, word=“usu-al”, hyphen=“u-su-al”) hyph.txt.en <-
correct.hyph(hyph.txt.en, word=“choices”, hyphen=“choi-ces”) hyph.txt.en <-
correct.hyph(hyph.txt.en, word=“ar-eas”, hyphen=“a-re-as”) hyph.txt.en <-
correct.hyph(hyph.txt.en, word=“Achieve”, hyphen=“A-chieve”) hyph.txt.en <-
correct.hyph(hyph.txt.en, word=“cir-cum-stances”, hyphen=“cir-cum-stan-ces”)
hyph.txt.en <- correct.hyph(hyph.txt.en, word=“items”, hyphen=“i-tems”) hyph.txt.en <-
correct.hyph(hyph.txt.en, word=“sim-p-ly”, hyphen=“simp-ly”) hyph.txt.en <-
correct.hyph(hyph.txt.en, word=“Again”, hyphen=“A-gain”) hyph.txt.en <-
correct.hyph(hyph.txt.en, word=“avail-a-ble”, hyphen=“a-vai-la-ble”) hyph.txt.en <-
correct.hyph(hyph.txt.en, word=“avail-a-ble”, hyphen=“a-vai-la-ble”) hyph.txt.en <-
correct.hyph(hyph.txt.en, word=“healthy”, hyphen=“heal-thy”) hyph.txt.en <-
correct.hyph(hyph.txt.en, word=“RE-SOURCES”, hyphen=“Re-sour-ces”) hyph.txt.en <-
correct.hyph(hyph.txt.en, word=“ser-vices”, hyphen=“ser-vi-ces”) hyph.txt.en <-
correct.hyph(hyph.txt.en, word=“places”, hyphen=“pla-ces”) hyph.txt.en <-
correct.hyph(hyph.txt.en, word=“in-terests”, hyphen=“in-ter-ests”) hyph.txt.en <-
correct.hyph(hyph.txt.en, word=“ac-ti-v-i-ties”, hyphen=“ac-ti-vi-ties”) hyph.txt.en <-
correct.hyph(hyph.txt.en, word=“able”, hyphen=“ab-le”) hyph.txt.en <-
correct.hyph(hyph.txt.en, word=“Able”, hyphen=“Ab-le”) hyph.txt.en <-
correct.hyph(hyph.txt.en, word=“or-di-nary”, hyphen=“or-di-nar-y”) hyph.txt.en <-
correct.hyph(hyph.txt.en, word=“equip-ment”, hyphen=“e-quip-ment”) hyph.txt.en <-
correct.hyph(hyph.txt.en, word=“around”, hyphen=“a-round”) hyph.txt.en <-
correct.hyph(hyph.txt.en, word=“re-quire”, hyphen=“re-qui-re”) hyph.txt.en <-
correct.hyph(hyph.txt.en, word=“dis-tances”, hyphen=“dis-tan-ces”) hyph.txt.en <-
correct.hyph(hyph.txt.en, word=“Emo-tion”, hyphen=“E-mo-tion”) hyph.txt.en <-
correct.hyph(hyph.txt.en, word=“even”, hyphen=“e-ven”) hyph.txt.en <-
correct.hyph(hyph.txt.en, word=“Re-quires”, hyphen=“Re-qui-res”) hyph.txt.en <-
correct.hyph(hyph.txt.en, word=“ca-nes”, hyphen=“canes”) hyph.txt.en <-
correct.hyph(hyph.txt.en, word=“class-ma-tes”, hyphen=“class-mates”) hyph.txt.en <-
correct.hyph(hyph.txt.en, word=“as-sistance”, hyphen=“as-sis-tance”) hyph.txt.en <-
correct.hyph(hyph.txt.en, word=“med-i-c-i-nes”, hyphen=“me-di-cines”) hyph.txt.en <-
correct.hyph(hyph.txt.en, word=“par-tic-i-pants”, hyphen=“par-ti-ci-pants”) hyph.txt.en
<- correct.hyph(hyph.txt.en, word=“Many”, hyphen=“Ma-ny”) hyph.txt.en <-
correct.hyph(hyph.txt.en, word=“Eval-u-at-ing”, hyphen=“E-val-u-at-ing”) hyph.txt.en <-
correct.hyph(hyph.txt.en, word=“un-pleasant”, hyphen=“un-pleas-ant”) hyph.txt.en <-
correct.hyph(hyph.txt.en, word=“adult”, hyphen=“a-dult”) hyph.txt.en <-
correct.hyph(hyph.txt.en, word=“emo-tion-al”, hyphen=“e-mo-tion-al”) hyph.txt.en <-
correct.hyph(hyph.txt.en, word=“dif-ficul-ties”, hyphen=“dif-fi-cul-ties”) hyph.txt.en <-
correct.hyph(hyph.txt.en, word=“ca-res”, hyphen=“cares”) hyph.txt.en <-
correct.hyph(hyph.txt.en, word=“re-al-ly”, hyphen=“real-ly”) hyph.txt.en <-
correct.hyph(hyph.txt.en, word=“night-ma-res”, hyphen=“night-mares”) hyph.txt.en <-
correct.hyph(hyph.txt.en, word=“di-ar-rhoea”, hyphen=“di-ar-rhoe-a”) hyph.txt.en <-
correct.hyph(hyph.txt.en, word=“re-st-ing”, hyphen=“res-ting”) hyph.txt.en <-
correct.hyph(hyph.txt.en, word=“itchy”, hyphen=“it-chy”) hyph.txt.en <-
correct.hyph(hyph.txt.en, word=“very”, hyphen=“ve-ry”) hyph.txt.en[1:91]

coleman.liau(pedsql_instructions.text, hyphen=hyph.txt.en)
dale.chall(pedsql_instructions.text, word.list="dale_chall_list_withplurals.txt")
flesch(pedsql_instructions.text, hyphen=hyph.txt.en)
flesch.kincaid(pedsql_instructions.text, hyphen=hyph.txt.en)
FOG(pedsql_instructions.text, hyphen=hyph.txt.en) FORCAST(pedsql_instructions.text, hyphen=hyph.txt.en) SMOG(pedsql_instructions.text, hyphen=hyph.txt.en)
